# OTME-6. Deep sequencing reveals heterogeneity of brain metastasis-associated macrophages and microglia and uncovers their cell type-specific functions within the tumor microenvironment

**DOI:** 10.1093/noajnl/vdab070.057

**Published:** 2021-07-05

**Authors:** Michael Schulz, Tijna Alekseeva, Julian Anthes, Jandranka Macas, Birgitta Michels, Aylin Möckl, Katja Niesel, Anna Salamero-Boix, Stefan Stein, Henner Farin, Karl H Plate, Yvonne Reiss, Franz Rödel, Lisa Sevenich

**Affiliations:** 1 Georg-Speyer-Haus, Frankfurt, Germany; 2 Biological Science Faculty, Goethe University, Frankfurt, Germany; 3 Institute of Neurology, University Hospital, Goethe University, Frankfurt, Germany; 4 Frankfurt Cancer Institute, Frankfurt, Germany; 5 German Cancer Consortium /German Cancer Research Center, Heidelberg, Germany; 6 Department of Radiotherapy and Oncology, Goethe University, Frankfurt, Germany

## Abstract

Macrophages represent a highly plastic cell type,indispensable for tissue and organ homeostasis, as well as innate immunity. Basic and translational research attributed tumor-promoting functions to macrophages, and their presence is often associated to poor patient prognosis and therapy resistance. While brain-resident macrophages, the so-called microglia (MG), represent the major immune cell type in the parenchyma under normal conditions, primary and metastatic brain tumors induce the recruitment of different immune cell types from the periphery, including monocyte-derived macrophages (MDM). Controversy remained about the redundancy of disease-associated molecular signatures and functions. The identification of markers that reliably distinguish brain-resident from blood-borne tumor-associated macrophages (TAMs) allowed the interrogation of molecular traits of different TAM populations in mouse and human brain tumors.

Using RNA-Seq, we demonstrated that TAMs rapidly acquire disease-associated transcriptional programs upon initial tumor infiltration, while gene expression remained stable during different stages of BrM progression. Across different BrM models, disease-associated transcriptional changes revealed lineage-specific, non-redundant functions of TAM populations, which was further reflected by cell type-specific occupation of different niches within the BrM microenvironment. Furthermore, we observed dose- and cell type-specific immune modulatory effects of whole brain radiotherapy on myeloid cells in BrM leading to a transient loss of disease-associated transcriptional programs predominately in blood-borne myeloid populations. This effect can at least in part be attributed to a replenishment of the recruited macrophage pool. This observation was further supported by scRNA-Seq analyses revealing higher heterogeneity of TAM-MDM compared to TAM-MG under treatment-naïve conditions and in response to radiotherapy.

Together, our results point towards the phenotypic plasticity of TAMs, especially MDMs, and the contribution of each compartment in instigating cancer-associated inflammation or the establishment of an immuno-suppressive TME. While TAM-MG exert functions related to pro-inflammatory responses, TAM-MDM are rather involved in tissue repair and regulation of adaptive immune cell functions.

